# Long-Term Outcomes of Radiation Therapy for Pediatric Brain Tumors: A Single-Center Study

**DOI:** 10.7759/cureus.81282

**Published:** 2025-03-27

**Authors:** Yukiko Fukuda, Masashi Endo, Kazunari Ogawa, Satoru Takahashi, Michiko Nakamura, Masahiro Kawahara, Keiko Akahane, Harushi Mori, Akira Gomi, Katsuyuki Shirai

**Affiliations:** 1 Department of Radiology, Jichi Medical University Hospital, Tochigi, JPN; 2 Department of Radiology, Jichi Medical University Saitama Medical Center, Saitama, JPN; 3 Department of Pediatric Neurosurgery, Jichi Children’s Medical Center Tochigi, Tochigi, JPN

**Keywords:** long-term outcomes, pediatric brain tumor, radiation therapy, radiotherapy (rt), single-center study

## Abstract

Brain tumors are the leading cause of mortality among pediatric patients. Recent advancements in genetic analysis have facilitated the development of new therapeutic agents, and high-precision radiotherapy techniques have improved survival rates for certain pediatric brain tumors. However, owing to the rarity of these tumors and the diversity of histological types, most treatment results are reported in clinical trials, and real-world data on the long-term treatment effects of radiotherapy in Japan are scarce. This study investigated the long-term outcomes of pediatric brain tumor treatment at a single institution. A total of 54 pediatric brain tumor patients aged ≤14 years who had undergone radiotherapy between 2007 and 2021 were included. Irradiation was performed using three-dimensional conformal or intensity-modulated radiation therapy. The distribution of each tumor type was as follows: eight diffuse intrinsic pontine gliomas, six malignant gliomas, 12 medulloblastomas, eight ependymomas, 15 germ cell tumors, and five other tumors (malignant peripheral nerve sheath tumor, pinealoblastoma, atypical teratoma/rhabdoid tumor, primitive neuroectoderm tumor, and malignant astroblastoma). The median follow-up duration for all patients and survivors was 48.4 months and 110 months, respectively. The one-, five-, and 10-year overall survival rates according to tumor type were as follows: diffuse intrinsic pontine glioma - 12.5%, 0%, and 0%; malignant glioma - 50%, 0%, and 0%; medulloblastoma - 91.7%, 83.3%, and 58.3%; ependymoma - 100%, 50%, and 37.5%; germ cell tumors - 93.3%, 93.3%, and 93.3%; and others - 80%, 80%, and 40%, respectively. The one-, five-, and 10-year progression-free survival rates according to the tumor type were 0% for both diffuse intrinsic pontine gliomas and malignant gliomas; 75%, 50%, and 50% for medulloblastoma; 62.5%, 25%, and not available for ependymoma; 86.7%, 80%, and 80% for germ cell tumor; and 60%, 40%, and not available for other tumors, respectively. Adverse events of grade 3 or higher (based on common terminology criteria for adverse events version 5.0) were observed in three patients as follows: two with hearing impairment and one with secondary cancer. Our findings revealed that the prognosis and recurrence patterns such as local and disseminated recurrence substantially differ depending on the tumor type. This confirms that each tumor type requires a unique approach. In recent years, significant progress has been made in the stratification and optimization of treatment through genetic analysis. However, to achieve improved tumor control and minimize late effects, the accumulation of long-term clinical data is essential.

## Introduction

Tumors of the central nervous system (CNS) account for approximately 20.0% of all childhood cancers, with an annual incidence of 2.5 to four cases per 100,000 children [1]. Treatment regimens for brain tumors vary according to tumor histology, location, and patient age [2,3]. Total surgical resection is considered prognostic for many tumor types [4]. However, the localization and invasiveness of the tumor can make resection difficult. Even with gross total resection, some tumor types have high local recurrence and dissemination rates. In such cases, radiation therapy is an effective treatment modality [5]. However, radiation therapy for pediatric brain tumors has several difficulties, such as lower tolerable doses to CNS tissues compared to adults, different histologic types, and the small number of cases due to the rarity of the disease, making it difficult to establish evidence-based on randomized controlled trials (RCTs) remains challenging [6,7].

With the development of molecular characterization, new drug therapies, and high-precision radiation therapy in recent years, clinical outcomes of certain pediatric brain tumors such as germ cell tumors, medulloblastoma, and ependymoma, have substantially improved [8]. In 2016, molecular characterization was integrated with traditional pathology, facilitating a targeted therapeutic approach for pediatric brain tumors. However, because pediatric brain tumors are rare and require long-term follow-up, available real-world data (RWD) in Japan are scarce. The inclusion of a highly selective patient population and the rigorously controlled conditions in RCTs may not be reflective of real-world clinical practice. Real-world evidence obtained from an analysis of RWD from observational studies can bridge gaps in evidence not addressed by RCTs [9]. RWD studies are increasingly being utilized as alternative sources of evidence in clinical cancer research [10]. In the case of rare diseases such as pediatric brain tumors, observational studies play an important role in identifying appropriate patient populations and clinical outcome assessments and biomarkers [11]. As mentioned above, the diagnostic criteria for pediatric brain tumors are changing significantly, and genetic information is becoming essential for pathological diagnosis. Although it is most important to diagnose brain tumors using the new pathological diagnostic criteria, determine the treatment plan, and evaluate the clinical outcomes, it will take several decades before long-term treatment results based on the new diagnostic criteria are reported. Therefore, there are significant gaps in the current situation where long-term outcomes and adverse events data for pediatric patients are most needed. We conducted a retrospective study because we thought it was important to present real-world data on the long-term prognosis of diseases diagnosed using the diagnostic criteria for pathological diagnosis that have been used in Japan to date. This study aimed to determine how prognosis and recurrence patterns differ depending on the tumor type and to identify the limitations and areas for improvement in current treatment.

## Materials and methods

Patient population

Pediatric brain tumor patients aged ≤14 years (n=55) who had undergone radiotherapy between 2007 and 2021 were selected from the database. Following the exclusion of one patient who had not undergone brain irradiation, 54 patients were included in the analysis.

Treatment protocol

Tumor type was determined by pathological diagnosis based on biopsy (n=43), imaging diagnosis was based on magnetic resonance imaging (MRI) (only eight patients of diffuse intrinsic pontine glioma), and clinical diagnosis was based on tumor markers (only three patients of germ cell tumor). Depending on the histological type and risk group, the treatment strategies included radiation therapy alone, surgical resection (including biopsy) plus radiation therapy, chemotherapy plus radiation therapy, or surgery plus chemotherapy plus radiation therapy. Radiotherapy was administered using three-dimensional conformal radiation therapy or intensity-modulated radiation therapy, and dose fractionation and irradiation fields were determined by discussion between pediatric neurosurgeons and radiation oncologists according to the tumor type and risk group.

Statistical analysis

The endpoints were one-, five-, and 10-year overall survival (OS), progression-free survival (PFS), and local control (LC). OS was defined as the time from the date of initiation of the first radiation therapy to the date of death from any cause, with surviving patients censored during the latest follow-up. PFS was defined as the interval between the date of the first radiotherapy and confirmed recurrence (local recurrence or dissemination {intracranial or spinal}, whichever occurred first) and was censored at the latest follow-up for patients who did not experience a recurrence. Local recurrence was defined as a recurrence within the irradiation field, including cases in which adequate local control was not achieved, and was determined using MRI images by a pediatric neurosurgeon and two radiation oncologists. LC time was calculated as the interval from the date of the first radiotherapy to the confirmation of local recurrence, censored at the latest follow-up for patients without local recurrence. Survival time analysis was performed using Kaplan-Meier survival curves, and differences between tumor types were compared between groups using the log-rank test. Data analysis was performed using EZR software version 1.68 (Tochigi, Japan: Yoshinobu Kanda) (2024-6-30) (http://www.jichi.ac.jp/saitama-sct/SaitamaHP.fles/statmedEN.html).

Ethical considerations

Written informed consent was obtained from all participants involved in the study or their parents. This study used an opt-out-consent approach. The study was conducted in accordance with the Declaration of Helsinki and approved by the Institutional Review Board of Jichi Medical University Hospital (#22-067).

## Results

Table 1 summarizes the baseline patient characteristics. This study included 54 patients (30 males and 24 females). The median age at irradiation for all patients was 9.0 years (range: 2-14). The tumor types were diffuse intrinsic pontine glioma (DIPG) (n=8 patients, 14.8%), malignant glioma (n=6, 11.1%), medulloblastoma (n=12, 22.2%), ependymoma (n=8, 14.8%), germ cell tumor (n=15, 27.8%), and others (n=5 patients, 9.3%), including malignant peripheral nerve sheath tumor, pineoblastoma, atypical malformation/rhabdoid tumor, primitive neuroectodermal tumor, and malignant astrocytoma (n=1 each). Based on the tumor type and risk, treatment strategies included radiation therapy alone in six patients, chemotherapy plus radiation therapy in six patients, surgical resection (including biopsy) plus radiation therapy in nine patients, and surgery plus chemotherapy plus radiation therapy in 33 patients. Craniospinal irradiation (CSI) was selected for medulloblastomas, germ cell tumors, and ependymomas with dissemination; whole-ventricle irradiation for germ cell tumors without dissemination; and local irradiation for ependymomas, DIPG, malignant gliomas, and other tumors without dissemination. The total dose and number of patients per irradiation site are summarized in Table 1. Of the 54 patients, 33 experienced recurrences (intracranial and/or spinal cord dissemination), and 16 received salvage radiotherapy.

**Table 1:** Baseline patient characteristics. CSI: craniospinal irradiation; WB: whole brain; WV: whole ventricle; boost: local boost to tumor or tumor bed

Total number, n (%)	54 (100)
Sex, n (%)	
Male	30 (55.6)
Female	24 (44.4)
Tumor type, n (%)	
Diffuse intrinsic pontine glioma	8 (14.8)
Malignant glioma	6 (11.1)
Medulloblastoma	12 (22.2)
Ependymoma	8 (14.8)
Germ cell tumor	15 (27.8)
Others	5 (9.3)
Therapy, n (%)	
Radiotherapy only	5 (9.3)
Chemotherapy + radiotherapy	6 (11.1)
Surgery + radiotherapy	10 (18.5)
Surgery + chemotherapy + radiotherapy	33 (61.1)
Irradiation field (total dose), n (%)	
CSI + boost (CSI 18.0-36.0 Gy, total 51.0-61.2 Gy)	19 (35.2)
Whole brain ± boost (WB 23.4-35.2 Gy, total 23.4-55.6 Gy)	3 (5.5)
Whole ventricle ± boost (WV 23.4-25.2 Gy, total 23.4-50.0 Gy)	9 (16.7)
Local irradiation (48.6-60.8 Gy)	23 (42.6)

**Figure 1:**
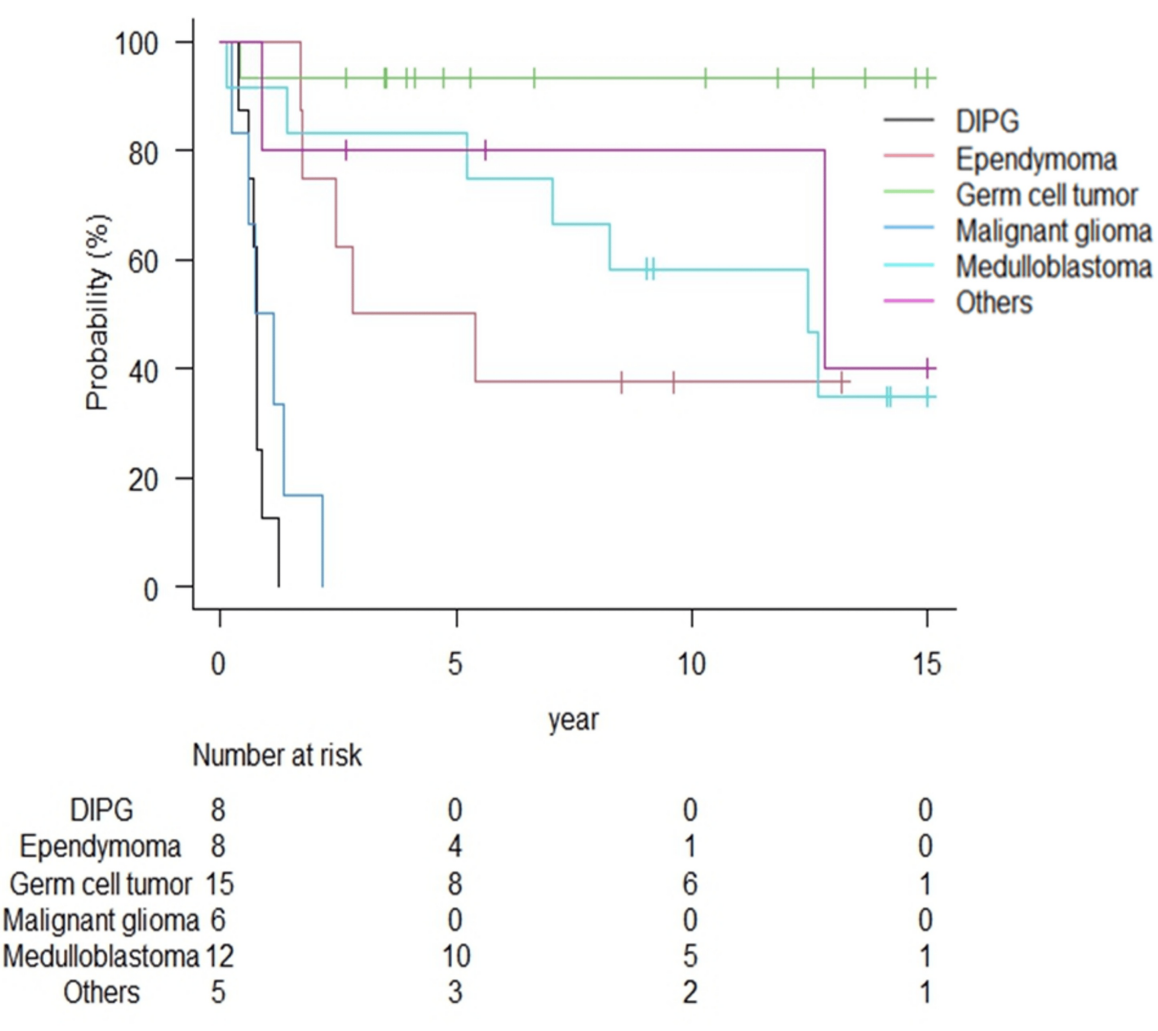
Overall survival rates according to tumor type. The five-year overall survival rates for DIPG and malignant glioma were both 0%; for germ cell tumors, there was only one death in 17 years; for medulloblastoma and ependymoma, the survival rate declined over time. DIPG: diffuse intrinsic pontine glioma

**Figure 2:**
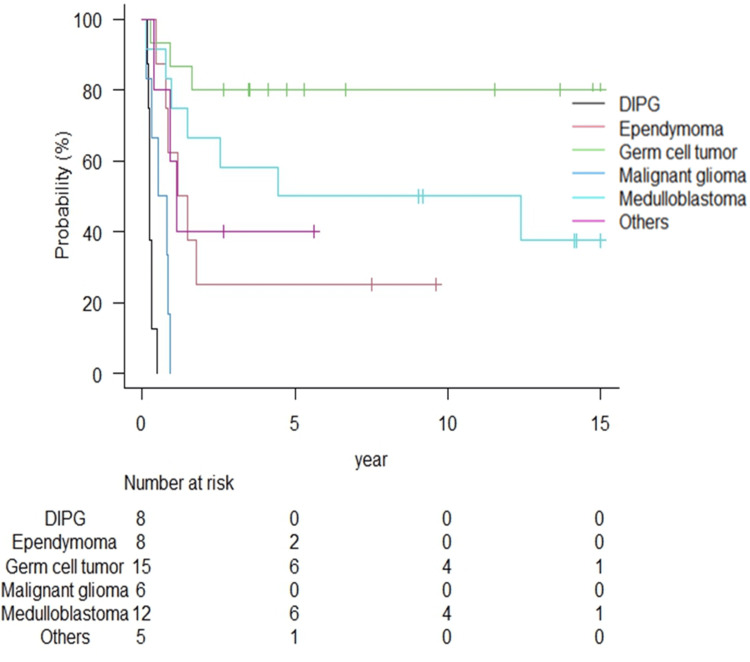
Progression-free survival rates according to tumor type. The one-year progression-free survival rates for DIPG and malignant glioma were both 0%. Recurrence of any tumor type often occurs within five years, with 75% of ependymomas experiencing recurrence within five years. DIPG: diffuse intrinsic pontine glioma

**Figure 3:**
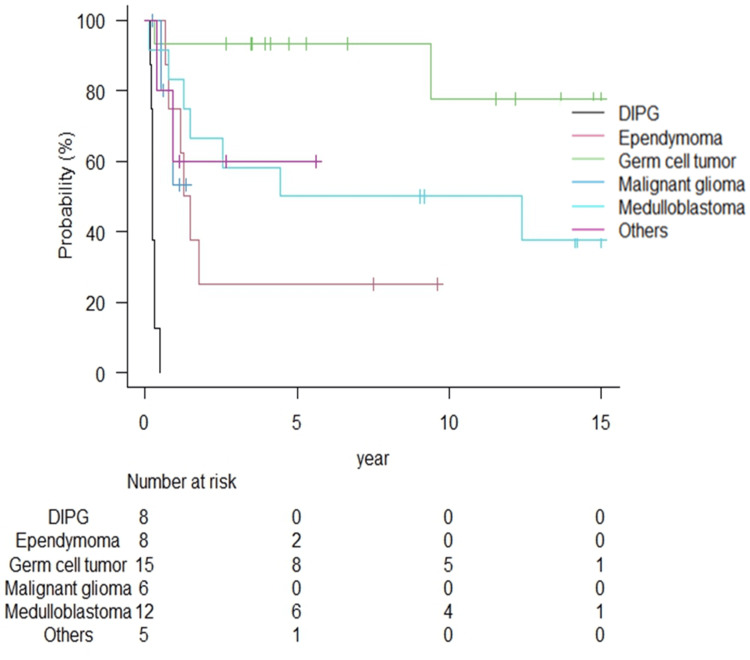
Local control rates according to tumor type. The one-year local control rates for DIPG and malignant glioma were both 0%, and the median duration of local control for DIPG was only 2.65 months. Local recurrence within five years was more common for medulloblastoma and ependymoma, while some patients experienced local recurrence after about 10 years for germ cell tumor. DIPG: diffuse intrinsic pontine glioma

Table 2 summarizes the radiation therapy outcomes for each tumor type. The median follow-up duration for all patients and survivors was 48.4 months (range: 1.3-192.5 months) and 110.0 months (range: 31.7-192.5 months), respectively. The Kaplan-Meier curves of OS, PFS, and LC according to tumor type are shown in Figures 1-3.

**Table 2:** Treatment outcomes. MST: median survival time (months); NA: not available

Tumor type	MST (months)	Overall survival (%)			Progression free survival (%)			Local control (%)		
		1 year	5 year	10 year	1 year	5 year	10 year	1 year	5 year	10 year
Diffuse intrinsic pontine glioma	9.1	12.5	0	0	0	0	0	0	0	0
Malignant glioma	11.1	50.0	0	0	0	0	0	0	0	0
Medulloblastoma	149.6	91.7	83.3	58.3	75.0	50.0	50.0	83.3	50.0	50.0
Ependymoma	33.6	100.0	50.0	37.5	62.5	25.0	25.0	75.0	25.0	25.0
Germ cell tumor	NA	93.3	93.3	93.3	86.7	80.0	80.0	93.3	93.3	77.8
Others	153.7	80.0	80.0	40.0	60.0	40.0	40.0	60.0	60.0	60.0

**Figure 4:**
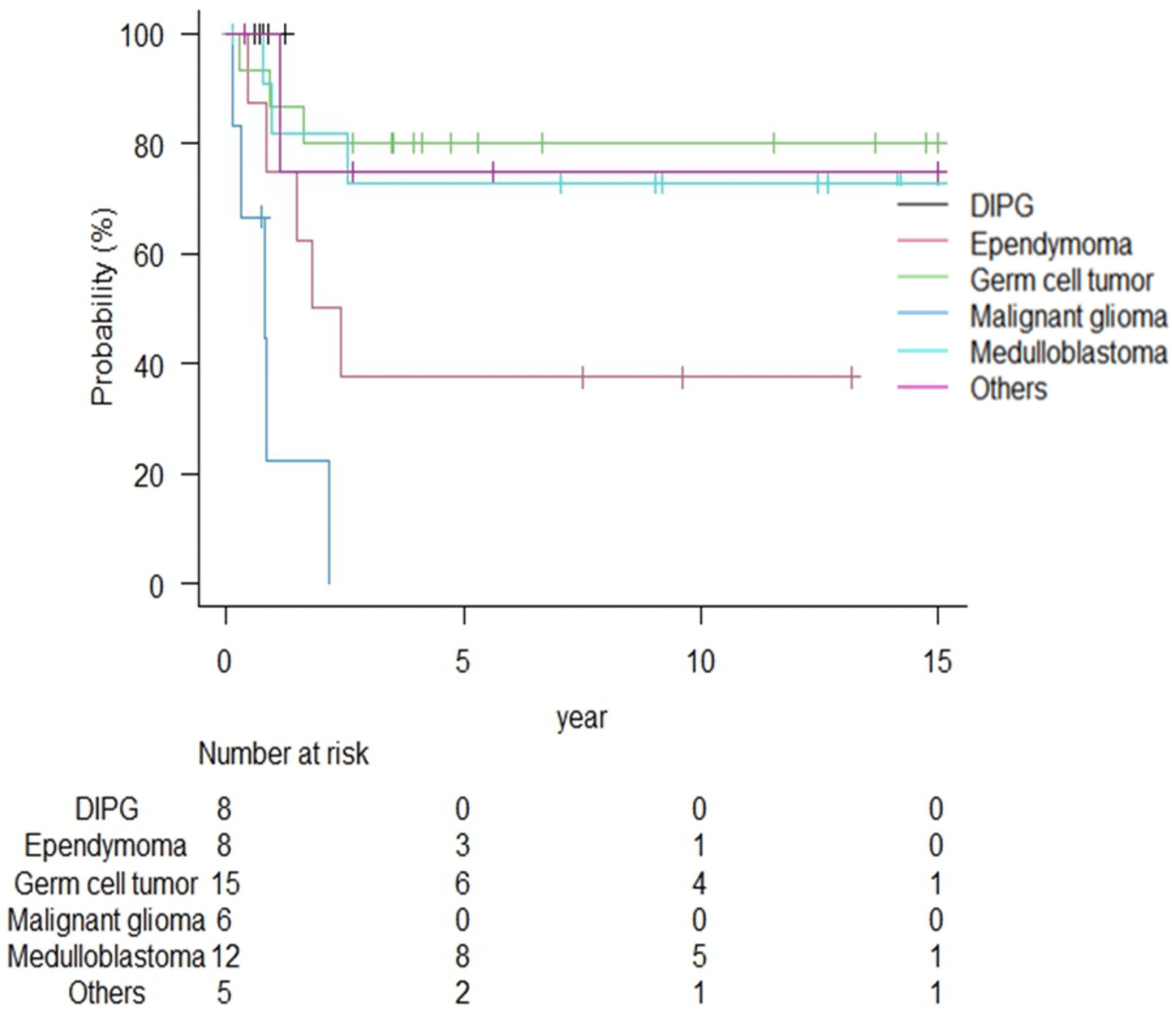
Dissemination recurrence according to tumor type. Dissemination includes intracranial and spinal cord. Dissemination was 0% in DIPG, whereas 83.3% of malignant gliomas resulted in dissemination at approximately two years. Dissemination in other tumor types all occurred within five years; however, as many as 62.5% of patients in ependymoma experienced dissemination, compared with an incidence of about 20% for medulloblastoma and germ cell tumor. DIPG: diffuse intrinsic pontine glioma

In addition, we evaluated the patterns of recurrence for each tumor type. Recurrence (local and/or dissemination recurrence {intracranial or spinal cord}) was observed in 33 of the 54 patients during the entire study period. Details of the recurrence types are shown in Table 3. Local recurrence was reported in all eight patients with DIPG, including one with intracranial dissemination. Only one patient with malignant glioma had local recurrence, and others had intracranial/spinal dissemination. Of the 12 patients with medulloblastoma, seven (58.3%) experienced recurrence, with only local recurrence in four and local recurrence plus dissemination (intracranial/spinal) in three patients. Six of the eight patients with ependymoma experienced recurrence, with only local recurrence and local recurrence plus dissemination (intracranial/spinal) in one and five patients, respectively. In patients with germ cell tumors, recurrence was observed in three patients, including intracranial, spinal cord dissemination, and local and spinal cord dissemination recurrence (n=1 each). The Kaplan-Meier curves for dissemination recurrence are shown in Figure 4. During the 17-year follow-up period, grade 3 or higher adverse events (Common Terminology Criteria for Adverse Events version 5.0) were reported in two patients, including one instance of hearing impairment and secondary cancer.

**Table 3:** Recurrence pattern according to tumor type. Local recurrence refers to the recurrence within the irradiation field, while dissemination involves intracranial recurrence outside the irradiation field or spinal recurrence. DIPG: diffuse intrinsic pontine glioma

Tumor type (n)	DIPG (8)	Malignant glioma (6)	Medulloblastoma (12)	Ependymoma (8)	Germ cell tumor (15)	Others (5)
Recurrence, n (%)	8 (100)	6 (100)	7 (58.3)	6 (75)	3 (20)	3 (60)
Local	7 (87.5)	1 (16.7)	4 (33.3)	1 (12.5)	0 (0)	1 (20)
Dissemination	0 (0)	4 (66.7)	0 (0)	0 (0)	1 (6.7)	2 (40)
Local + dissemination	1 (12.5)	1 (16.7)	3 (25)	5 (62.5)	2 (13.3)	0 (0)

## Discussion

With the development of genetic diagnostics, new therapeutic agents, improved surgical techniques, and advances in radiation therapy, the five-year survival rate for childhood cancer has increased to approximately 80.0% [8]. However, prognosis varies depending on the tumor type and other risk factors. Our findings indicated significant differences in long-term outcomes according to the tumor type. Although this series predates the incorporation of genetic diagnoses, we present real-world data from our institution and discuss the long-term outcomes and recurrence patterns for each tumor type.

DIPG

The most lethal subtype of high-grade glioma in children is diffuse median line glioma, which typically occurs in the thalamus and/or brainstem [12]. Eighty percent of diffuse midline gliomas occur in the cerebral cortex and are known as DIPG [13]. Given their specific localization, surgical resection remains extremely challenging, and biopsies are avoided unless the clinical or imaging findings are atypical [14]. To date, no chemotherapy regimen has demonstrated efficacy for DIPG [15,16], and local radiation therapy remains the mainstay treatment [17]. However, the outcomes of the standard treatment using 54 Gy in 27 fractions of local irradiation were not satisfactory. Hoffman et al. reported that the two and five year OS rates of patients with DIPG were 10% and 2%, respectively, and that the median survival time was 11 months [13]. In the present study, all patients were diagnosed based on MRI findings, without histological biopsy. Local irradiation (range: 48.6-60.8 Gy) resulted in a one-year OS in 12.5%, five-year OS in 0%, and median survival time (MST) of 9.1 months, corroborating the findings of other studies. The median duration of LC and PFS was 2.7 months, with all patients experiencing local recurrence, including one with intracranial dissemination. These results indicate that standard local radiation therapy provides only temporary symptomatic relief; however, increasing the local dose to 72-78 Gy does not contribute to improvement in PFS and OS, highlighting the limitation of radiation therapy for tumor control [18]. Furthermore, regarding salvage therapy at recurrence, re-irradiation of patients who responded well to initial radiotherapy demonstrated improved clinical and radiological response while minimizing the risk of acute toxicity. However, re-irradiation does not contribute to OS prolongation, underscoring the critical need for developing new therapeutic strategies for tumor control [19]. From a radiotherapy perspective, diffuse midline glioma remains a highly lethal tumor with a poor prognosis. Hypofractionated irradiation (e.g., 39 Gy in 13 fractions or 45 Gy in 15 fractions) may offer a promising alternative by reducing the duration of treatment by increasing the one-line dose, maintaining comparable efficacy to those of conventional fractionated methods, thereby reducing patient burden and could be a promising treatment option [20,21]. In recent years, the H3K27M mutation has been identified in this disease, and various new treatment strategies, such as molecular-targeted drugs and CAR-T therapy, are being researched. While radiation monotherapy, which is the current standard treatment, is also essential, it does not provide sufficient tumor control, so it is thought that the development of a treatment method that combines radiation and drugs is necessary for the future outlook.

Malignant glioma

Among pediatric malignant gliomas, glioblastoma (GBM) is characterized by distinct genetic subtypes and is considered biologically and prognostically different from adult GBMs. In particular, pediatric GBM has a very low incidence of isocitrate dehydrogenase mutations, with proven resistance to temozolomide [15]. Conversely, pediatric GBM may have a better prognosis than adult GBM [22,23]. The optimal treatment strategy for pediatric GBM has not yet been established and generally involves total resection followed by radiation therapy. A large cohort study involving 1173 pediatric GBMs reported an MST of 15 months, a one-year OS of 58%, and a five-year OS of 17% [24]. In the present study, four of six patients with malignant glioma had GBM, with an MST of 11.1 months, one-year OS of 50%, and five-year OS of 0%. In the present study, all six patients developed recurrence, one with local recurrence only, one with spinal cord recurrence only, and the remaining four with local and dissemination (intracranial or spinal cord) recurrence. Extracranial metastases at diagnosis are present in less than 2% of adult malignant gliomas [25]. Given their rarity, spinal cord MRI and CSF cytology are not routinely performed at initial diagnosis. Local recurrence often causes treatment failure [26]. In the present study, the one-year incidence of dissemination was significantly higher for medulloblastomas (18.2%), ependymomas (25%), and malignant gliomas (77.8%; p<0.001). The high incidence of dissemination recurrence, despite the short survival period, could be attributed to the difficulty in achieving LC, suggesting that malignant gliomas in children may be more prone to dissemination than those in adults. Advances in genetic and molecular research are revealing that malignant glioma (particularly glioblastoma) in adults and children has very different characteristics, but we believe that elucidating the pathology of this disease is a challenge in order to develop more effective treatment strategies.

Medulloblastoma

Medulloblastoma is a relatively common malignant brain tumor in children that generally arises in the posterior cranial fossa. Treatment of medulloblastoma followed the principle of maximal surgical resection combined with CSI and chemotherapy. Traditionally, treatment has been stratified based on clinical features, such as the extent of the residual tumor after resection and the presence of metastases. Recent advances in genetic profiling have identified molecular subtypes that correlate with prognosis, with a five-year OS of 85% at average risk and 70% at high risk [27]. In Japan, the five-year OS rate for the SJMB-96 regimen was 80.8% at mean risk and 74.2% at high risk [28]. In this study, 50% of the patients had intracranial or spinal cord metastatic disease at diagnosis classifying them as high-risk (n=5); however, the overall five-year OS was 83.3%, five-year PFS was 50%, and MST was 149.6 months, consistent with those of previous studies. The gross total resection group (n=5) had a significantly better prognosis (p=0.002), whereas the group with initial dissemination (n=6) had a significantly worse prognosis (p=0.03).

Recent studies have stratified medulloblastomas into a low-risk group with a good prognosis (wingless-type {WNT}, low-risk sonic hedgehog {SHH}, low-risk group 3/4) and a high-risk group with a poor prognosis (high-risk SHH, high-risk group 3/4) [27]. Ongoing clinical trials are investigating the potential for radiation dose reduction based on risk stratification; if the initial dose of CSI can be reduced, irradiation-induced late toxicity will be reduced [29]. In our study, recurrence was observed in seven patients (58.3%) during the course of the disease, four of whom had local recurrences only, and the remaining three had local and spinal cord dissemination recurrences. No intracranial dissemination was observed in any of the patients. This suggests that treatment failure is often due to local recurrence and that local dose reduction in future treatment protocols should be done cautiously. Personalized therapies, such as targeted therapy and immunotherapy, based on the molecular profiles of subgroups, are being explored, warranting further clinical studies to validate these new treatment modalities [30].

Ependymoma

Ependymomas, which originate from the ependymal cells lining the ventricles, are serious malignancies of the central nervous system, with more than 50% of patients experiencing recurrence [31]. The 2021 revised WHO classification of brain tumors emphasizes genetic diagnosis in addition to localization and histopathologic features [32]. Although genetic mutations associated with prognosis have been identified, no treatment options have been directly linked to these findings. Clinical trials demonstrating the benefit of chemotherapy to ependymoma survival are scarce. Chemotherapy improves survival outcomes by promoting tumor shrinkage, which in turn may increase the likelihood of achieving gross total resection. The standard of care is complete surgical resection followed by 54-59.4 Gy of local radiation therapy. The largest randomized controlled trial reported a five-year OS and PFS of 86.2% and 68.5%, respectively, in 479 pediatric patients with ependymomas who underwent complete resection [33,34]. The incidence of dissemination at initial diagnosis is low (2.2%), and CSI was not performed in patients without initial dissemination [34]. Our study findings indicated a poor prognosis, with an MST of 33.6 months, a five-year OS of 50%, and a five-year PFS of 25%. This discrepancy may be attributed to the fact that all included patients had anaplastic ependymomas, which are associated with a poor prognosis. Additionally, MST was significantly shorter in the group with intracranial/spinal cord dissemination at the time of initial treatment (n=2) than in the group without dissemination (n=6; p=0.02). Furthermore, recurrence was reported in six out of eight patients, with only one reported local recurrence and the remaining experiencing local and dissemination recurrence. Four of the local recurrences were within the irradiated field (30-59.4 Gy), and two were in areas receiving less than 30 Gy. These results suggest that the current doses are insufficient for tumor control. In addition, CSI should be considered at the time of initial treatment for high-risk cases because the recurrence of spinal cord dissemination is not infrequent, even if dissemination is not obvious at the time of the initial disease. Achieving complete tumor control in ependymomas remains challenging owing to the tolerable dose in the brain and spinal cord, highlighting the need for developing effective drug therapies.

Germ cell tumor

Germ cell tumors (GCTs) are classified as embryomas or non-germinomatous germ cell tumors (NGGCTs) based on their histological composition and degree of differentiation. Germinomas are more common in older children, whereas NGGCTs occur primarily in younger children [35]. Germinomas are highly sensitive to chemotherapy and radiation therapy, with a five-year OS rate of >90% [35]. A complete response rate of 84% has been reported with chemotherapy alone, and salvage radiation therapy is effective against relapse [36]. In contrast, NGGCTs are less sensitive to radiation, and optimal treatment varies widely depending on the histological characteristics of the tumor with no established standard of care [37]. Calaminus et al. reported a five-year OS rate of 67% for patients with NGGCT after scientific radiotherapy [38]. In the present study, 11 of the 15 GCTs were germinomas, and four were mixed germ cell tumors. The five-year OS and PFS were 93.3% and 80%, respectively. The five-year OS rate for germinomas was 100%, whereas that for mixed germ cell tumors was 75%, consistent with previous reports. Four of the germinomas had intracranial or spinal cord dissemination at initial presentation; however, the presence or absence of dissemination did not affect the prognosis. Tumor localization did not contribute to OS, PFS, LC, or incidence of dissemination. Recurrence was observed in three patients as follows: intracranial, spinal cord dissemination, and local and spinal cord dissemination recurrence (n=1 each). All recurrences were treated with salvage radiotherapy, and there was only one case of primary mortality. Because of the good long-term survival of germ cell tumors, clinical trials are currently underway to reduce the radiation dose with the aim of reducing late adverse events [39]. The trial also investigates localized irradiation up to 54 Gy without CSI for non-disseminated malignant NGGCT. However, as the NGGCT group also showed a higher incidence of recurrence involving the spinal cord than the germinoma group in this analysis, the question of whether it is acceptable to omit CSI at the time of initial treatment remains, and we await the results. However, NGGCTs include diverse histopathologic types, and their sensitivity to chemotherapy and radiotherapy may vary. Therefore, collecting information on tumor characteristics and prognosis is crucial to establishing effective treatment strategies.

Limitations

This study had several limitations. First, this was a retrospective study with a small number of cases. However, it is important to present real-world data with long-term follow-up. Second, there are various confounding factors in the clinical outcomes of pediatric brain tumors, and they are extremely complex, including factors such as age, tumor type, tumor location, presence or absence of dissemination, radiation dose and irradiation range, tumor type, and treatment method, etc.; however, it was not possible to adjust for these factors and secure the necessary number of cases to obtain highly reliable data, so it was not possible to perform multivariate analysis in this analysis. Third, some older patients did not have detailed histology because genetic analysis was not performed. Although we cannot rule out the possibility of crossover owing to the complex histology of pediatric brain tumors, the long-term results suggest that our diagnosis was reasonable.

## Conclusions

The results of long-term radiation therapy for pediatric brain tumors at our institution were generally comparable to those previously reported in other studies. The findings of this study revealed that the prognosis and recurrence patterns such as local and disseminated recurrence substantially differ depending on the tumor type. This confirms that each tumor type requires a unique approach. In the future, stratification and optimization of treatments based on genetic analysis will continue to advance. Furthermore, advancements in the field of radiotherapy continue to evolve, aiming for further tumor control and reduction of late effects.
